# Advances on Epigenetic Drugs for Pediatric Brain Tumors

**DOI:** 10.2174/1570159X20666220922150456

**Published:** 2023-05-18

**Authors:** Panagiotis Skouras, Mariam Markouli, Dimitrios Strepkos, Christina Piperi

**Affiliations:** 1 Department of Biological Chemistry, Medical School, National and Kapodistrian University of Athens, 11527 Athens, Greece

**Keywords:** Pediatric brain tumors, pediatric gliomas, medulloblastoma, ependymoma, epigenetics, epigenetic drugs, drug repurposing, DNMTi, HDACi

## Abstract

Pediatric malignant brain tumors represent the most frequent cause of cancer-related deaths in childhood. The therapeutic scheme of surgery, radiotherapy and chemotherapy has improved patient management, but with minimal progress in patients’ prognosis. Emerging molecular targets and mechanisms have revealed novel approaches for pediatric brain tumor therapy, enabling personalized medical treatment. Advances in the field of epigenetic research and their interplay with genetic changes have enriched our knowledge of the molecular heterogeneity of these neoplasms and have revealed important genes that affect crucial signaling pathways involved in tumor progression. The great potential of epigenetic therapy lies mainly in the widespread location and the reversibility of epigenetic alterations, proposing a wide range of targeting options, including the possible combination of chemo and immunotherapy, significantly increasing their efficacy. Epigenetic drugs, including inhibitors of DNA methyltransferases, histone deacetylases and demethylases, are currently being tested in clinical trials on pediatric brain tumors. Additional novel epigenetic drugs include protein and enzyme inhibitors that modulate epigenetic modification pathways, such as Bromodomain and Extraterminal (BET) proteins, Cyclin-Dependent Kinase 9 (CDK9), AXL, Facilitates Chromatin Transcription (FACT), BMI1, and CREB Binding Protein (CBP) inhibitors, which can be used either as standalone or in combination with current treatment approaches. In this review, we discuss recent progress on epigenetic drugs that could possibly be used against the most common malignant tumors of childhood, such as medulloblastomas, high-grade gliomas and ependymomas.

## 1 INTRODUCTION

Pediatric Brain Tumors (BTs) present the most common cause of death in children due to their pathology, clinical complications and resistance to therapy [1]. A striking example of therapeutic complications is in medulloblastoma (MB) treatment, which is often accompanied by neuroendocrine and neurocognitive impairment [2, 3]. Pediatric BTs typically arise in different locations, often hindering the application of radiation therapy, which may negatively impact brain development, especially in patients under five years old [4].

BTs were originally classified based on their histological profile. Advances in genomic sequencing, however, have led to the revelation of genetic and epigenetic changes that point out the importance of molecular profiling [5]. In this context, the most recent Central Nervous System 5 (CNS5) World Health Organization (WHO) classification has divided gliomas,glioneuronal and neuronal tumors into 6 different groups, distinguishing the adult-type diffuse gliomas, which comprise Isocitrate Dehydrogenase (IDH)-wild type glioblastomas (GBs), the pediatric-type diffuse low-grade and diffuse high-grade gliomas, the circumscribed astrocytic gliomas, the glioneuronal and neuronal tumors and the ependymomas [4]. Tumors not falling under these categories are represented as separate categories, such as embryonal tumors, which include MBs.

Interestingly, the new classification for gliomas, glioneuronal and neuronal tumors has incorporated two new groups of pediatric tumors: pediatric-type diffuse low-grade and pediatric-type diffuse high-grade gliomas. This change in tumor classification reflects the prognostic profile of each group and encompasses the molecular work-up required to fully characterize each tumor. Accordingly, all pediatric-type low-grade gliomas are expected to have a better prognosis and usually exhibit overlapping histological features. This group consists of 4 types of tumors: Diffuse astrocytoma, MYB- or MYB-Like 1 (MYB-L1)-altered, Angiocentric glioma, Polymorphous low-grade neuroepithelial tumor of the young and Diffuse low-grade glioma, Mitogen-Activated Protein Kinase (MAPK) pathway-altered. On the other hand, pediatric-type diffuse high-grade gliomas tend to have a worse prognosis. They consist of the Diffuse midline glioma (DMG) Histone 3 (H3) lysine 27 (K27) altered (including the tumors known as Diffuse Intrinsic Pontine Gliomas, DIPGs) already described in the 2016 classification, as well as 3 new other types of tumors: diffuse hemispheric glioma H3 Glycine 34 (G34)-mutant, diffuse pediatric-type high-grade glioma H3- and IDH-wild type, as well as infant-type hemispheric glioma. Of note, the term GB is no longer used to describe any pediatric-type tumors.

Molecular changes are crucial for the characterization as well as the better understanding of the pathology of each pediatric tumor. These molecular alterations have been shown to directly impact gene expression and interact with other regulatory mechanisms to fine-tune gene expression.

Among these mechanisms, epigenetic modifications have attracted scientific interest to bridge each tumor's phenotypic features with the expression of key genes. Epigenetic alterations serve as molecular mechanisms to alter the expression of specific genes without directly altering the DNA sequence. Epigenetic mechanisms include DNA methylation and histone modifications such as methylation, acetylation, ubiquitinylation, demethylation and deacetylation, and non-coding RNAs (ncRNAs) [6]. Histone Post-Translational Modifications (PTMs) mostly occur on the histone amino-terminal tails protruding from nucleosomes’ surface and modulating gene expression by altering chromatin structure and recruiting transcriptional regulators [7, 8]. They can cause chromatin condensation, which does not allow transcriptional enzymes to bind to gene sequences, thus decreasing their expression. Alternatively, they can cause chromatin “relaxation,” which enhances the binding of transcriptional enzymes to genetic loci and increases their expression [9]. In this way, they provide an extra reversible regulatory step in gene expression and enable the cell to tightly control specific genes that will be upregulated or downregulated.

There is evidence that epigenetics play a major role in the characterization and pathology of brain tumors. Some tumors have key mutations which harbor an epigenetic impact, like the case of pediatric-type diffuse high-grade gliomas, where the H3K27 mutation (H3K27M) and H3G34-mutant variants demarcate the distinct tumor subgroups in the latest WHO classification. In this context, DNA methylation profiling, when interpreted in the setting of clinical, histopathological, radiological, and other molecular data, can enable the diagnosis of CNS tumors to a great extent, especially in cases when conventional immunohistochemistry is insufficient, or genetic and morphological features may be inconclusive. Molecular profiling is an extremely valuable tool that can provide a wide variety of information, including tumor classification, family and subfamily types, single gene promoters and the methylation status of DNA copy number variants [10].

The field of epigenetics further provides a promising opportunity for the future management of pediatric brain tumors. Epigenetic tumor profiling has allowed identifying critical changes in tumor development, establishing a more accurate tumor classification and prediction of disease prognosis, and selection of new potential epigenetic drug targets [11]. The advantage of epigenetic drugs relies on their reversibility and the lower risk of side effects. Another important parameter is the potential for Convection-Enhanced Delivery (CED), allowing the compound to be directly delivered to the tumor without further side effects [12] to overcome the obstacle of the Blood Brain Barrier (BBB).

Currently, a vast array of therapeutic epigenetic-based approaches is being tested in clinical trials. In more detail, epigenetic drugs have already been studied in various cancer types, such as hematological malignancies. Clinical trials for pediatric brain tumors have also been initiated, hoping to address resistance to current treatment options. Epigenetic profiling of these tumors has already been presented as a novel clinical tool expected to complement standard therapies and improve the clinical trial design with more suitable patient recruitment [5].

This review discusses the pathological characteristics of the most common pediatric brain cancers and provides the latest advances in epigenetic profiling, epigenetic drug targeting, and treatment options.

## 2 MAIN TYPES OF PEDIATRIC BRAIN TUMORS

The most common malignant brain tumor subtype in children is MB, followed by ependymomas and high-grade gliomas that include DMGs and DIPGs [5]. Clinical trials investigating the use of epigenetic drugs against these three tumor subtypes are currently being carried out and discussed in the following sections.

### 2.1 Medulloblastoma (MB)

MB is a frequent and extensively studied pediatric cerebellar tumor. The latest classification of MB highlights the significant role of molecular characteristics in their diagnosis and has correlated them with histopathological features [4]. The molecular subgroups of MB include the WNT-activated, the Sonic Hedgehog (SHH)-activated, and the group 3 and 4 MB. SHH-activated MBs are further divided into Tumor Protein 53 (TP53)-mutated and TP53-wild type tumors. Analysis of their epigenome has also revealed 4 different subgroups in the SHH-activated MB and 8 subgroups in the non-WNT and non-SHH MB, which provide clinical utility [13-16]. These different subtypes of MB are associated with varying degrees of response to treatment, including tumor resection, craniospinal radiation and chemotherapeutic agents [17, 18]. Therefore, the overall survival rate of MB patients in the last decade has been around 70% [19], with WNT-activated MB patients exhibiting a better prognosis. On the other hand, patients with group 3 MBs harboring *MYC* amplification and SHH MBs with *TP53* mutations or *MYCN* amplification have a significantly less favorable prognosis with a 5-year progression-free survival of less than 50% [13].

Epigenetic changes have been crucial in differentiating the different subtypes of MB. In more detail, EZH2 overexpression, increased H3K27me3 methylation and decreased H3K4 methylation marks account for most alterations found in groups 3 and 4 MB [20]. Combined with the epigenetic regulation of WNT through various pathways, such as the Hippo/YAP pathway [21] and the epigenetic role of SHH, which induces activation of bivalent genes [22], the importance of epigenetic modifications in the different MB subtypes becomes even more evident.

The histopathological features of MB are similar to those in the 2016 classification and include the classic, the desmoplastic/nodular, the MB with increased nodularity and the large cell/anaplastic MB. Each one of the MB subtypes has a unique expression and clinical phenotype [23, 24]. The combination of histopathological features with the molecular and epigenetic changes observed in MB provides a more accurate picture of tumor characteristics, helps to define prognosis, and selection of the most effective therapy.

Regarding children with the WNT-activated subtype of MB, 95% will survive past the first 5 years after diagnosis [25]. Beta-catenin and monosomy 6 are biomarkers and characterize the prognosis of the WNT-activated subtype and the majority of the MB subtypes [26]. The adherens junction Catenin Beta 1 (*CTNNB1*) gene has been shown to exhibit a variety of mutations in the WNT subtype [27, 28] while it encodes for beta-catenin, which induces the transcription of WNT target genes [29]. Moreover, it has been shown that H3K4me3 is enriched in actively expressed genes and associated with molecular changes in many types of brain tumors, including MB [30].

### 2.2 High-Grade Gliomas (HGGs)

The expected 5-year survival of patients with HGGs is less than 5%, and children with DIPG have a life expectancy of less than a year [1, 31]. DIPGs and other H3K27M-mutated DMGs are universally lethal pediatric CNS tumors. DIPG tumors are tightly connected to mutations in the H3 Histone, Family 3A (*H3F3A*) gene. This gene encodes the H3.3 histone, and its mutation results in the H3K27M [32, 33]. The H3K27 trimethylation (H3K27me3) in H3K27M leads to the inhibition of the N-methyltransferase EZH2 (Enhancer of Zeste Homolog-2), which is an imperative subunit of PRC2 (Polycomb Repressive Complexes-2) [34, 35]. Mutations in histone encoding genes (H3F3A, Histone Cluster 1 H3 Family Member B (HIST1H3B)) resulting in lysine substitution H3K27M are key drivers of early gliomagenesis [36]. PRC2 is a regulator of specific genes related to tumor suppression or oncogenes. Lewis *et al.* proposed a model of glioma progression through epigenetic silencing, namely H3K27M-mediated inhibition of PRC2 activity. The alteration of H3K27me3 to H3K27M inhibits the PRC2 complex. Additionally, H3K27M leads to the accumulation of H3K27ac, which is related to active transcription [36] and results in the formation of heterotypic nucleosomes, H3K27M-H3K27 acetylation (H3K27ac) [37, 38]. Lastly, heterotypic nucleosomes recruit Bromodomain-containing proteins [39].

It is important to mention that HGGs can be divided into 2 subgroups, the K27 and the G34 subgroup, based on the mutations of genes that encode histone 3 (*H3F3A, HIST1H3B/C*), the IDH subgroup and the H3 or IDH wild-type subgroup [1]. The oncohistone H3.3K27M co-occurs with *TP53* mutations and enhancement of PDGFRA (Platelet Derived Growth Factor-Alpha), whereas H3.1K27M coexists with mutations related to genes that participate in the PI3K (Phosphatidylinositol 3-Kinase) signaling pathway and mutations in Activin receptor type-1 (*ACVR1*) [40-42].

DMGs are often located in the brainstem, thalamus, or spinal cord. Moreover, DIPGs account for almost 80% of all pediatric tumors of the brainstem and are characterized as the deadliest tumor, with only 1% of the children surviving 5 years after the diagnosis [43], due to its intrinsic nature, which makes it impossible to be surgically resected [44, 45]. DIPGs are also unresponsive to chemotherapy, while radiotherapy is minimally effective [45, 46]. The 80% of midline pontine HGGs bear the H3K27M mutation, which is crucial for tumor progression [33]. Mutations in H3.1 and H3.3 histones have been detected in DMG and DIPG tumors [40, 47].

### 2.3 Ependymoma (EPN)

Ependymoma classification is also based on molecular alterations and histopathological characteristics. Moreover, the anatomical position of ependymomas is crucial in their characterization. The 3 anatomical locations for ependymomas as per 2021 WHO classification involve the supratentorial area where ependymomas can be sub-grouped in Zinc Finger Translocation Associated (ZFTA) fusion-bearing or Yes Associated Protein 1 (YAP-1) fusion-bearing, the posterior fossa where ependymomas are divided in Posterior Fossa A (PFA) and PFB groups, and the spinal compartments where ependymomas are observed to have an *MYCN* amplification [4]. EPNs exhibit a high frequency in children [44] and are mostly (90%) found intracranially, frequently in the hindbrain. Some EPNs may also arise in the posterior fossa but are extremely rare in the spine [48].

Regarding the therapeutic options of this subtype, 40% of the tumors remain incurable [49, 50], and the mechanisms related to resistance to chemotherapy have yet to be elucidated. The 10-year overall survival of pediatric patients is 64% with a variable clinical phenotype [51]. Some patients have a slow disease progression, followed by recurrence, a few years later, while others exhibit a rapidly progressing clinical course [52]. Concerning infants with EPN, the 5-year survival is estimated to be 42-55% [53]. Chemotherapies have been proven ineffective against EPN [50], and surgery, combined with radiation, is the main therapeutic approach, improving event-free survival [54, 55].

Depending on the tumor's anatomical location (Supratentorial/Posterior Fossa-hindbrain), there appears to be heterogeneity based on the molecular subtypes of EPNs [56]. Intracranial EPN is characterized by a dysregulation of FGFR (Fibroblast Growth Factor Receptor) and EGFR (Epidermal Growth Factor Receptor) [49, 56-58]. Regarding the PF EPN group, there are two sub-groups: PF-EPN group A, found in young patients with the fatal disease [59] and PF-EPN group B, characterized by a milder course [1, 59]. PF-EPN-A, compared to PF-EPN-B, exhibits a CpG island methylator phenotype (CIMP). Another category of EPN is the Supratentorial (ST) EPN, with a methylated profile and poor prognosis [49, 60].

Global H3K4me3 levels are often associated with the modification of genes such as Cyclin D1 (*CCND1*) and Erb-B2 Receptor Tyrosine Kinase 2 (*ERBB2*), which are linked to therapeutic resistance in EPNs [56, 61]. *CCND1* and *ERBB2* have been characterized as EPN oncogenes [57, 62]. CIMP+ hindbrain EPNs appear sensitive to agents that target enzymes responsible for the H3K27me3 genetic alteration [48]. Reduction of H3K27me3 is seen in some PF-EPN cases, suggesting a strong outcome predictor for EPNs [63]. Lewis *et al.* demonstrated that H3K4me3 immunohistochemical (IHC) staining in PF-EPN-A tumors accurately predicts malignancy [61].

## 3 EPIGENETIC DRUGS

The significant heterogeneity between different tumor subtypes and within each tumor itself highlights the importance of personalized therapy, which could be achieved through epigenetic profiling and targeted epigenetic drugs. These drugs act on enzymes and proteins in epigenetic modifications [5].

The great potential of epigenetic therapy lies in the reversibility of epigenetic changes, which allows the functional recovery of epigenetically altered genes with normal DNA sequences, meaning that cancer cells can be reprogrammed back to a more physiologic state [64]. Even though their use as monotherapy in solid tumors may have limited selectivity compared to hematologic malignancies, limited solubility and sometimes unfavorable toxicity [65], the latest research has unveiled ways of solving these issues to benefit from the widespread targeting options that these drugs have to offer. In more detail, epigenetic therapies elicit robust responses, so reduced or intermittent dosing could be considered. Nanoscale delivery systems have also been shown to improve drug stability, increase permeability and cellular uptake, and achieve targeting specificity [66]. Moreover, their combination with other epigenetic drugs, chemo- or immunotherapy can help increase their therapeutic efficacy while allowing reduced drug dosing and unwanted side effects [66].

Current mechanisms of action for these drugs focus on inhibiting epigenetic enzymes, such as histone demethylases, deacetylases, and DNA methyltransferases, as well as inhibiting proteins that participate in epigenetic modifications, such as BET proteins, CDK9, AXL, FACT, BMI1, and CBP.

### 3.1 Jumonji Domain-containing 3 (JMJD3)/Ubiquitously Transcribed Tetratricopeptide Repeat, X Chromosome (UTX) Demethylase Inhibitors

A potential therapeutic approach for pediatric HGGs (pHGGs) is based on the restoration of the repressive H3K27me3 levels either by employing the inhibition of H3K27me3 demethylases and/or the Lysine Demethylase 6 (KDM6) subfamily of JMJD3 K27 demethylases [67]. The experimental drug GSK-J4 has been shown to inhibit JMJD3 in pediatric brainstem gliomas in K27M mutant cells *in vitro* and in K27M xenografts *in vivo*. It decreased cell viability by enhancing the S-phase of the cell cycle and apoptosis and reducing clonogenic growth [68]. Another study administered the GSK-J4 compound in patient-derived DIPG xenografts and demonstrated its beneficial effects in repairing DNA by homologous recombination while increasing tumor cell radiosensitivity [69]. APR-246, which targets p53 mutant proteins, was shown to enhance the radiosensitizing effect of GSK-J4 and result in the accumulation of reactive oxygen species [69, 70].

### 3.2 Histone Deacetylase Inhibitors (HDACi)

HDACi is a well-studied class of epigenetic modifiers. A pan-HDACi, Panobinostat, demonstrated promising results in preclinical evaluations for DIPG and is currently tested in a trial for children with DIPG (NCT02717455). There are some concerns about the ability of Panobinostat to cross the BBB [71]. However, it was shown to normalize the expression of genes affected by H3K27M and restore the levels of H3K27me3 [72], showing a high potency against pHHGs with an immediate influence on cell viability. H3K27M cells developed resistance [72] and did not exhibit any survival benefits in animal tumor models compared to controls after prolonged treatment [73]. Increased H3K27ac, in response to panobinostat, appears to take place mainly at the H3.3 histone variants and not at H3.1 or H3.2 [37]. It is important to mention that panobinostat suppresses leptomeningeal seeding (a rare complication of MB), which induces inflammation in the CNS in a mouse model [74]. Based on the importance of cellular epigenetic alterations due to histone modifications, HDACi, such as Valproic acid and Suberoylanilide hydroxamic acid (SAHA), have also been studied on a wide variety of tumors [75, 76]. In the study of Mack *et al.*, a combination of Decitabine (DAC) and an HDAC inhibitor, SAHA (FDA approved), was employed against PFA-CIMP+ EPN *in vitro* and *ex vivo*, and they were shown to exhibit additive effects compared with DAC mono-therapy [48]. A study by Halsall *et al.* showed that HDAC inhibition increases H3K27me3 in non-H3K27M expressing tumors of human lymphoblastoid cell lines, derived from B-lymphocytes immortalized by Epstein Barr Virus [77], and PRC2 restored its activity in H3K27M tumor poly-acetylated H3 tails [78]. Preclinical studies demonstrated that HDAC inhibitors eradicate DMG cells by reducing proliferation and repressing tumor growth *in vitro* and *in vivo* [72, 79, 80]. HDACi showed a promising interaction with pHGGs by decreasing the survival of DIPG cells in orthotopic xenograft models [72, 79]. Many HDACs have been tested in pHGGs, including vorinostat, entinostat, and panobinostat (LBH589), either as monotherapy or in combination with other agents, including Lysine Specific histone Demethylase 1 (LSD1) Inhibitors like Compound 7 [79], and others [37, 80-82].

### 3.3 DNA Methyltransferase Inhibitors (DNMTi) - EZH2 Inhibitors

Treatment of PFA-CIMP+ (CpG island methylator phenotype positive) cultures with DAC resulted in the de-repression of gene complexes containing EZH2 targets [48]. DNA Methyltransferase inhibitors (DNMTi) (such as 5-azacitidine) are in open trials for group A EPNs, which are characterized by hypermethylation [83]. DNMTi combined with immunotherapy is being investigated to improve efficacy [6, 83, 84]. EZH2 inhibitors aim to overcome the dysregulation of PRC2, which impacts cellular differentiation and proliferation [36]. EZH2 is the methyltransferase subunit of PRC2. This type of inhibitor was initially clinically tested in atypical teratoid tumors and rhabdoid tumors and may also prove to be a successful therapeutic approach for other tumors, such as group 3 and 4 MB and other gliomas (NCT02601937). Small-molecule EZH2 inhibition decreased cell viability and proliferation and showed prolonged survival in the DIPG mouse model (H3K27M tumors) through a mechanism dependent on the induction of the tumor-suppressor protein p16^INK4A^.

3-deazaneplanocin A (DZNep) degrades PRC2 complex proteins and diminishes trimethylation of H3K27 [85]. Treatment of PFA-CIMP+ EPN with DZNep *in vitro* showed lower expression of EZH2 and trimethylation of H3K27 [48]. *In vivo* treatment (flank model or orthotopic intracerebellar xenografts) with DZNep, of human PFA-CIMP+ EPN resulted in decreased tumor volume and improved survival [48].

### 3.4 Lysine Specific Histone Demethylase 1 (LSD1) Inhibitors

LSD1, also known as Lysine Demethylase 1A (KDM1A), has been detected as an important biologically validated epigenetic target for cancer therapy. The mechanism behind its regulation is the removal of the mono- and dimethyl group from H3K4/K9. LSD1 demethylates Lysine 4/9 at H3 histone (H3K4me1/2 and H3K9me1/2) by binding to the promoter regions and repressing or activating gene transcription [86]. LSD1 also carries a structural resemblance with Monoamine Oxidases (MAOs). Several inhibitor drugs, such as CC-90011, INCB059872, IMG-7289, and GSK-2879552, can target LSD1 [87] and have been tested as a therapy against acute myeloid leukemia, neuroblastoma and sarcoma [88]. H3K4me1 histone was detected to be enriched in pHGG cells [89], suggesting that LSD1 may impact the enhancers of genes playing a key role in pHGG pathophysiology. Furthermore, LSD1 inhibitors hinder demethylation by targeting its catalytic domain. SP2509 is a non-competitive and potent LSD1 inhibitor with anti-proliferative and anti-cancerous properties [90]. It promotes apoptosis in Ewing sarcoma cells by inducing Endoplasmic Reticulum (ER) stress response and subsequently inhibits cell proliferation [91, 92]. Combinatorial treatment of SP2509 and Panobinostat exerted cytotoxic effects in acute myeloid leukemia [91].

### 3.5 Bromodomain and Extra Terminal (BET) Inhibitors

BET proteins (Bromodomain Containing 2 and 4) can be recruited by acetylation of H3K27 and subsequently engage transcriptional co-factors, but also activate RNA Pol II-dependent transcription [39, 93]. Oncohistone H3K27M forms heterotypic nucleosome complexes with H3K27ac, which subsequently recruit Bromodomain Containing 2 and 4, indicating an additional therapeutic target (*i.e*., JQ1, I-BET151). These inhibitors decrease H3K27ac and reduce cell viability in DIPG [89]. Upon systemic administration of JQ1 in preclinical animal models, JQ1 was shown to cross the BBB [89]. Furthermore, JQ1 and I-BET151 induced differentiation decreased proliferation and tumor growth in mouse DIPG xenograft models. JQ1, compared to GSK-J4 inhibitor, was shown to be more effective on JMJD3 H3K27 demethylases [89]. Other studies also outlined JQ1 as a potential target for treating DIPG tumors with the Cyclin Dependent Kinase 7 (CDK7) inhibitor THZ1, targeting RNA Pol II phosphorylation [94, 95]. However, JQ1 demonstrated no significant toxicity in H3K27M cells compared to H3-wild-type cells [68]. Finally, BET bromodomain inhibitors also appear to play a role in pre-clinical MB models. The BET inhibitor JQ1 decreases cellular viability, causes G1 arrest and apoptosis of *MYC*-amplified MB cell lines, and downregulates *MYC* transcription and MYC targets [96, 97]. It further prolongs survival by decreasing tumor growth in cerebellar orthotopic MB models *in vivo* [98] and also induces cellular senescence, simultaneously suppressing transcriptional processes linked to poor prognosis in MB patients [99]. Moreover, JQ1 disrupts protein interactions leading to aberrant HH signaling, decreasing viability and proliferation of SHH-driven MB cell lines. The BET inhibitor I-BET151 also appears to significantly decrease HH activity [100] while decreasing MB viability and growth both *in vitro* and *in vivo*.

### 3.6 CDK9 Inhibitors

CDK9 is essential for maintaining gene silencing at heterochromatic loci [101]. Therefore, CDK9 inhibition causes the reactivation of epigenetically silenced genes in cancers, restoring the expression of tumor suppressor genes and cellular differentiation. Specifically, CDK9 inhibition results in the dephosphorylation of the SWI/SNF protein BRG1, which then contributes to the reactivation of gene expression [101].

Moreover, Dahl *et al.* studied the importance of AF4/FMR2 Family Member 4 (AFF4) in DIPG tumors [102]. AFF4 protein was key in the Super Elongation Complex (SEC) structure and was shown to maintain the clonogenic potential and promote self-renewal of DIPG tumors. Since CDK9 interferes with SEC, CDK9 inhibitors, Atuveciclib and AZD4573, were shown to block the release of RNA Pol II, inducing the expression of pro-differentiation genes in DIPG cells and also enhancing their self-renewal [103, 104]. Additionally, the CDK9 inhibitors delayed tumor growth, increased survival and demonstrated therapeutic benefits in orthotopic xenograft models of DMG [102].

### 3.7 AXL Inhibitors

Biopsies of DIPG exhibited increased AXL kinase expression, which was correlated with the presence of H3K27M. AXL expression has also been shown to be controlled by epigenetic regulators. More specifically, EZH2 has been shown to sustain AXL expression independently of DNA and histone methylation in glioblastoma cells [105]. Furthermore, YAP1 acts as a positive regulator of AXL expression in various cancers [106-108]. These findings add to the involvement of AXL in epigenetic pathways, demonstrating that AXL could serve as an alternative target in regulating the expressional response to epigenetic changes. Based on these data, BGB324 (an AXL-specific inhibitor) was tested in DIPG cells and was shown to affect the expression of epithelial differentiation markers by increasing their levels and downregulating the expression of mesenchymal genes [109]. It is worth mentioning that this AXL inhibitor was able to cross the BBB after oral administration and systemic administration in preclinical animal models [109].

### 3.8 Facilitates Chromatin Transcription (FACT) Inhibitors

FACT complex was revealed as a prominent target in pHGGs bearing H3K27M since one of its subunits interacts with the mutant histone H3.3K27M [82]. FACT has been shown to play a role as a histone chaperone and has been implicated in DNA repair, DNA replication, and transcription [109, 110]. The proteins Structure-Specific Recognition Protein 1 (SSRP1) and Suppressor of Ty16 (SPT16), which belong to this complex, have been overexpressed in DIPG tumors compared to normal brain tissues [82]. CBL0137 (a FACT inhibitor) reduced the survival of DIPG cells and decreased the growth of DIPG xenografts in mice. In addition, CBL0137 enhanced the trimethylation and acetylation of H3K27M [82] and systemic administration of this drug was shown to cross the BBB in preclinical animal models.

### 3.9 BMI1 Inhibitors

A member of the PRC1 complex, BMI1, is a chromatin remodeler that monoubiquitinates H2AK119 [111]. It was found to be increased in tumors with H3K27M along with the H2AK119Ub histone mark, compared to normal pons cells [112]. PTC209 and PTC028 (BMI1 inhibitors) inhibited the proliferation of DIPG cells and upregulated the expression of *p16* and *p21* tumor suppressor genes. However, inhibition of BMI1 resulted in Senescence Associated Secretory Phenotype (SASP) activation, increasing the probability of tumor relapse [112].

### 3.10 CBP Inhibitors

ICG-001, the structural inhibitor of the CBP acetyltransferase, blocks the association of CBP with other proteins. ICG-001 reduces cell survival, migration, invasion and radio-resistance in DIPG cells [113]. Other novel inhibitors, such as PDGFR and CDK4/6, have also proven to be effective against DIPG cells [114]. A recent study of GSK2830371 (a PPM1D inhibitor) in DIPG demonstrated that this inhibitor could sensitize DIPG cells through PARP inhibition [115].

## 4 COMBINATION THERAPY USING EPIGENETIC AGENTS

**Fig. (1):**
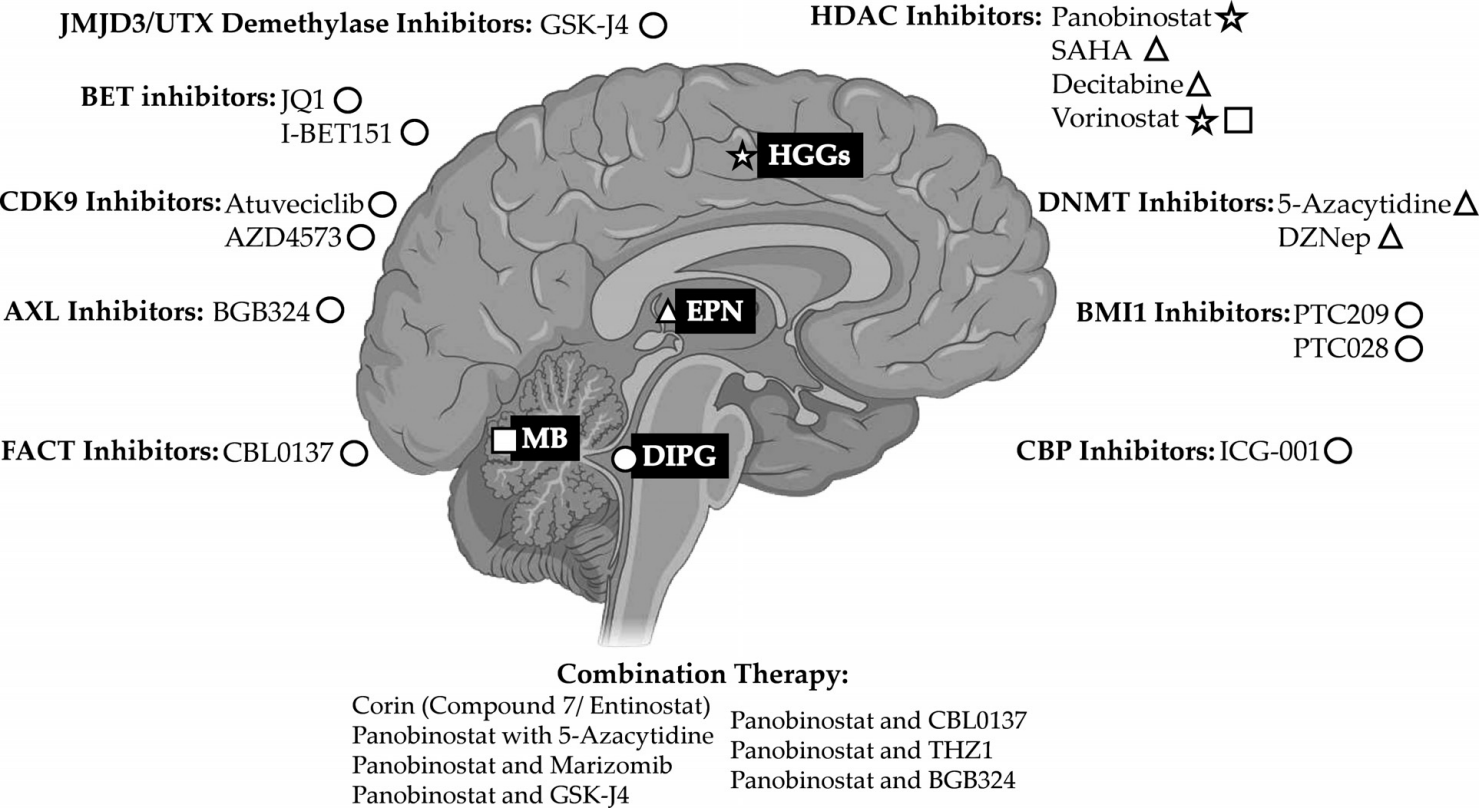
Epigenetic Drugs and suggested location of action. The schematic representation shows the location of pediatric tumor subtypes and the localized activity of the different epigenetic drugs. Notably, most drugs in the same category target the same tumor except for HDAC inhibitors which have a different tumor target. Notably, Decitabine has the same location of action as SAHA. Finally, combining different drug categories results in synergistic action against a tumor target (symbols: star for HGGs, triangle for EPN, square for MB, circle for DIPG). Created by BioRender.com (2022).

Combination therapy of conventional drugs with innovative epigenetic treatments has been shown to significantly improve therapeutic effects. Epigenetic drugs may be combined with chemotherapy to increase chromatin accessibility to chemotherapeutic drugs *via* chromatin decompaction [116]. Similar effects are observed when epigenetic drugs are combined with immunotherapy [117], possibly by re-expressing tumor-surface antigens and proteins of the major histocompatibility complex and re-activating endogenous retroviruses, tumor perceptibility by the immune system can be increased [118, 119]. Interestingly, epigenetic drugs can influence cancer and immune cells to achieve immunopotentiation and enhance antitumor responses [120]. Epigenetic drugs may therefore be combined with existing antitumor treatment options to help overcome acquired drug resistance of cancer cells and also help achieve immunopotentiation (Fig. **1**).

### 4.1 Repurposing Drugs

Drug repurposing, or drug repositioning, refers to uncovering new uses for existing drugs. Since resistance to conventional therapy in pediatric brain tumors is common, causing high mortality and poor patient survival, drug repurposing has emerged as a promising approach to identify novel treatment regimens, aiming to overcome resistance to therapy and successfully treat pediatric CNS tumors.

One example includes DAC (FDA-approved DNA demethylating agent), which is already being used for treating hematopoietic malignancies. It has been proposed as a repurposing drug in a clinical trial for children with CIMP+ PF-EPN group A [121]. Additionally, other FDA-approved drugs, vorinostat, romidepsin, and belinostat, have been used to treat T-cell lymphomas, and preclinical studies are currently exploring their efficacy on MB [122].

### 4.2 Current Evidence on Epigenetic Drug Combination in Adult and Pediatric Brain Tumors

LSD1 and HDAC inhibition have been proposed as a combination therapy that induces cell death in adult GB cells and patient-derived glial stem cells [123]. Anastas *et al.* employed a combinatorial therapeutic approach of a single molecule inhibitor, Corin (combination of Compound 7 and Entinostat), inhibiting HDAC and LSD1. Corin was administrated intracranially with CED in xenografts; it induced proliferation and cell survival [79]. Corin modified gene expression and induced the differentiation of H3K27M cells, unlike the treatment with the administration of Entinostat and Compound 7 separately [79].

Another therapeutic scheme, using panobinostat and GSK-J4, showed that their combination had adequate efficacy in low doses compared to monotherapies (single agent administration) [72], but further elucidation of the underlying molecular changes of this combination therapy is needed. Moreover, a combination of Panobinostat with THZ1 (CDK7 inhibitor) sensitized DIPG cells resistant to Panobinostat by targeting their transcriptional activity. On the contrary, the combination of panobinostat and JQ1 did not yield similar results [72, 94]. Azacytidine, with systemic administration, crosses the BBB in preclinical animal models [124]. However, 5-azacytidine with panobinostat improved the survival of mice with H3K27M tumors, compared to panobinostat as a single agent [37]. CBL0137 (FACT inhibitor) impaired the growth of xenografts and reduced the survival of DIPG cells in mice, synergizing with panobinostat [82]. A synergy of panobinostat and marizomib (Proteasome inhibitors) was shown to modify gene transcription in pHGG cells, particularly due to cytotoxic effects and induction of oxidative stress [81]. Of importance, systemic administration of marizomib was shown to cross the BBB in preclinical animal models [81]. Furthermore, combining BGB324 and panobinostat reduced cell proliferation in H3K27M cells but not in wild-type H3 histone cells. Additionally, these compounds synergized in inducing migration and invasion of DIPG cells. They also reversed the mesenchymal phenotype of cells by decreasing the expression of different genes such as Zinc Finger E-Box Binding Homeobox 1 (*ZEB1*), Zinc Finger E-Box Binding Homeobox 2 (*ZEB2*), Snail Family Transcriptional Repressor 2 (*SNAI2*), SRY-Box Transcription Factor 2 (*SOX2*) and Nestin (*NES*). Of note, the synergy of BGB324 and panobinostat enhanced the sensitization of DIPG cells to radiotherapy. Moreover, when combined and delivered through CED in mouse models of DIPG, there was a delay in tumor growth [109].

In another study, there was an induction of TRAIL-dependent cell death by combining the effects of EZH2 and HDAC inhibition when giving EPZ-6438 (EZH2i) and vorinostat with ONC201/TIC10 [125]. In more detail, ONC201 is a small molecule that crosses the BBB and acts against GB tumor cells and cancer stem cells (CSCs) by antagonizing the dopamine receptors DRD2 and 3. It is currently being evaluated in phase 2 clinical trials in patients with recurrent GB or H3 K27M high-grade gliomas (NCT02525692).

Another study employing a combination treatment of JQ1 and EPZ6438 injected in mice's primary Neural Stem Cell (NSC) cultures demonstrated inhibition of tumor growth, compared to the single use of each agent. This drug combination has a substantial impact on the tumor suppressor gene p16^INK4A^ but also the reduction of H3K27me3 levels [125].

The cytotoxic and anti-self-renewal effects of JQ1 and ICG-001 require preclinical testing in animal models and subsequently in clinical trials [113]. Balakrishnan *et al.* combined the PTC028 inhibitor with the BH3 mimetic Obatoclax, which binds to BCL2 proteins, inducing cellular apoptosis. This combinatorial treatment had a strong effect in inhibiting the growth of H3K27M pHGG cells, increasing the survival of mouse models compared to monotherapy and preventing SASP reprogramming, which increases the prospect of tumor recurrence [112]. Obatoclax was shown to cross the BBB in preclinical animal models when administered systemically [112] (Table **1**).

## CONCLUSION AND FUTURE PERSPECTIVES

Throughout the last decade, pediatric neuro-oncology has experienced significant advances in understanding childhood brain tumors through the analysis of genomic and epigenomic alterations, which pointed out their heterogeneity and enabled the identification of key molecular modifications associated with each tumor subtype. Identification of genomic or epigenomic alterations has enabled the prediction of patient disease outcomes, *i.e*., the prognosis of WNT-MB and poor outcomes in G3-MB, SHH-MB bearing the *TP53* mutation. In PF-EPN group A. Genomic functional studies have also improved our knowledge of oncogenic drivers. Future research may help develop animal models for studying different types of brain tumors and their biology.

However, the molecular alterations of G4-MB, PF-EPN groups A and B have yet to be clearly understood. Similarly, gene alterations that affect tumor progression need to be studied for G3-MB, ST-EPN-RELA and histone mutant HGG tumors. These examples suggest that a significant portion of tumorigenic genes remains unknown and requires further research to elucidate. There have been major advances in some subgroups of pediatric BTs (*i.e*., MB) but not in other HGG subtypes, where prognosis remains largely unaltered. Therefore, the extensive heterogeneity of HGGs needs to be thoroughly investigated to better characterize each tumor subgroup and determine patient-specific (personalized) therapies.

Molecular analysis of pediatric BTs has already revealed mutations in SMO antagonists or PTCH in patients with SHH-MB. Additional candidate therapies have been proposed, such as pemetrexed/gemcitabine and HDAC/PI3K inhibitors for G3-MB. It has been anticipated that PF-EPN group A and HGGs, which carry histone mutations, will respond to epigenetic regulators.

The combination of agents should also be extensively investigated through experimental studies and preclinical testing, especially in tumors with a single mutation, *i.e*., pilocytic astrocytomas, to increase efficacy. In this context, the combination of molecular-targeted therapies with immunotherapy should be explored. In molecular therapies for pediatric brain tumors, correct tumor classification is an imperative prerequisite due to the tumor-selective inhibition of molecular targets so that molecular patterns of healthy cells are not affected. In the case of incorrect patient selection, therapies may be ineffective and subsequently wrongly evaluated. Another potential treatment approach is the synergy of molecular-targeted therapies and immunotherapy regarding pediatric BTs. Furthermore, the acute and long-term sequelae of molecular-targeted therapies require careful monitoring since potential interference with the biological processes of healthy cells is always possible.

Overall, it is evident that pediatric brain tumors are characterized by lower mutational and neoantigen loads and different epigenetic profiles from those observed in adult brain tumors, indicating the need for further research. Therefore, a deeper understanding of the tumor microenvironment and signaling mechanisms that define each tumor subtype is crucial for developing even more effective personalized treatment strategies for CNS tumors in children. Recent efforts towards this direction have been promising, with epigenetic tumor profiling allowing for better tumor classification, more accurate patient selection for each therapeutic approach, and many clinical trials currently investigating potential treatment options for pediatric CNS tumors. Several molecular-targeted therapies acting on genetic and epigenetic modifications of pediatric brain cancers have already been developed. It is, however, important to specify the safety, selectivity, exact mechanism of action and BBB permeability of each of these drugs, but also predict patient outcomes using genomic, transcriptomic, and epigenetic data to select the least toxic and most effective treatment option for each patient.

## Figures and Tables

**Table 1:** Current clinical trials of epigenetic drugs in pBTs.

**S. No.**	**NCT Number**	**Title**	**Conditions**	**Interventions**	**Mechanism of Action of ** **the Epigenetic Drug**
**Histone Deacetylase Inhibitors (HDACis)**					
1	NCT04897880	A Study of Panobinostat in Pediatric Patients with Solid Tumors Including MRT/ATRT	-Recurrent Brain Tumor, Childhood -Rhabdoid Tumor -Atypical Teratoid/ Rhabdoid Tumor -Malignant Rhabdoid Tumor	Panobinostat	Panobinostat inhibits class I (HDACs 1, 2, 3, 8), class II (HDACs 4, 5, 6, 7, 9, 10) and class IV (HDAC 11) proteins
2	NCT04341311	Phase I Study of Marizomib + Panobinostat for Children With DIPG	-Diffuse Intrinsic Pontine Glioma -Pediatric Brainstem Glioma -Pediatric Brainstem Gliosarcoma, Recurrent -Pediatric Cancer -Pediatric Brain Tumor -Diffuse Glioma	-Drug: Marizomib -Drug: Panobinostat	Panobinostat inhibits class I (HDACs 1, 2, 3, 8), class II (HDACs 4, 5, 6, 7, 9, 10) and class IV (HDAC 11) proteins
3	NCT03893487	Fimepinostat in Treating Brain Tumors in Children and Young Adults	-Diffuse Intrinsic Pontine Glioma -Recurrent Anaplastic Astrocytoma -Recurrent Glioblastoma -Recurrent Malignant Glioma -Recurrent Medulloblastoma	-Drug: Fimepinostat -Procedure: Therapeutic Conventional Surgery	Fimepinostat inhibits the activity of both PI3K class I isoforms and HDAC
4	NCT03566199	MTX110 by Convection Enhanced Delivery in Treating Participants with Newly Diagnosed Diffuse Intrinsic Pontine Glioma	Diffuse Intrinsic Pontine Glioma	-Drug: Panobinostat Nanoparticle Formulation MTX110 -Drug: Convection Enhanced Delivery (CED)	Panobinostat inhibits class I (HDACs 1, 2, 3, 8), class II (HDACs 4, 5, 6, 7, 9, 10) and class IV (HDAC 11) proteins
5	NCT02717455	Trial of Panobinostat in Children With Diffuse Intrinsic Pontine Glioma	Glioma	-Drug: LBH589 (Panobinostat)	Panobinostat inhibits class I (HDACs 1, 2, 3, 8), class II (HDACs 4, 5, 6, 7, 9, 10) and class IV (HDAC 11) proteins
6	NCT02420613	Vorinostat and Temsirolimus With or Without Radiation Therapy in Treating Younger Patients with Newly Diagnosed or Progressive Diffuse Intrinsic Pontine Glioma	Diffuse Intrinsic Pontine Glioma	-Other: Laboratory Biomarker Analysis -Radiation: Radiation Therapy -Drug: Temsirolimus -Drug: Vorinostat	Vorinostat inhibits the enzymatic activity of histone deacetylases HDAC1, HDAC2 and HDAC3 (Class I) and HDAC6 (Class II)
7	NCT01189266	Vorinostat and Radiation Therapy Followed by Maintenance Therapy With Vorinostat in Treating Younger Patients with Newly Diagnosed Diffuse Intrinsic Pontine Glioma	-Anaplastic Astrocytoma -Anaplastic Oligoastrocytoma -Diffuse Intrinsic Pontine Glioma -Gliosarcoma	-Radiation: 3- Dimensional Conformal Radiation Therapy -Radiation: Intensity Modulated Radiation Therapy -Other: Laboratory Biomarker Analysis -Drug: Vorinostat	Vorinostat inhibits the enzymatic activity of histone deacetylases HDAC1, HDAC2 and HDAC3 (Class I) and HDAC6 (Class II)
8	NCT00879437	Valproic Acid, Radiation, and Bevacizumab in Children With High-Grade Gliomas or Diffuse Intrinsic Pontine Glioma	-Glial Cell Tumors -Malignant Gliomas -Glioblastoma Multiforme -Anaplastic Astrocytoma -Gliomatosis Cerebri -Gliosarcoma -Brainstem Glioma -Diffuse Intrinsic Pontine Glioma	-Drug: Valproic acid -Drug: Bevacizumab -Radiation: Radiation therapy	Valproic acid inhibits class I (HDAC1, HDAC2, HDAC3, HDAC8) and class IIa (HDAC4, HDAC5, and HDAC7) HDACs
9	NCT02265770	An International Clinical Program for the Diagnosis and Treatment of Children With Ependymoma (SIOP-EP-II)	Childhood ependymoma	Stratum III: -Chemotherapy: Vincristine Carboplatin Methotrexate Cyclophosphamide Cisplatin Valproate -Drug: Valproate	Valproate inhibits class I (HDAC1, HDAC2, HDAC3, HDAC8) and class IIa (HDAC4, HDAC5, and HDAC7)
10	NCT03838042	INFORM2 Study Uses Nivolumab and Entinostat in Children and Adolescents With High-risk Refractory Malignancies (INFORM2 NivEnt)	- CNS Tumor, Childhood -Solid Tumor, Childhood	-Drug: Nivolumab –Drug: Entinostat	Entinostat is a highly selective histone deacetylase 1 (HDAC1) and HDAC3 inhibitor
**DNA Methyltransferase Inhibitors (DNMTs)**					
11	NCT03206021	COZMOS: Phase I/Ib Trial of Combined 5'Azacitidine and Carboplatin for Recurrent/Refractory Pediatric Brain/Solid Tumors (COZMOS)	-Recurrent Childhood CNS Tumor -Ependymoma, Recurrent Childhood -Childhood Solid Tumor	-Chemotherapy: carboplatin -Drug: 5-Azacytidine	5-Azacytidine gets incorporated into the DNA and irreversibly binds to DNA methyltransferases, thus inhibiting their activity
**Bromodomain and Extra Terminal (BET) Inhibitors**					
12	NCT03936465	Study of the Bromodomain (BRD) and Extra-Terminal Domain (BET) Inhibitor BMS-986158 in Pediatric Cancer	-Brain Tumor, Pediatric -Solid Tumor, Childhood -Lymphoma	Drug: BMS-986158	BMS-986158 is an inhibitor of the Bromodomain BET family of proteins
**BMI1 Inhibitors**					
13	NCT03605550	A Phase 1b Study of PTC596 in Children with Newly Diagnosed Diffuse Intrinsic Pontine Glioma and High-Grade Glioma	-High-Grade Glioma -Diffuse Intrinsic Pontine Glioma	-Drug: PTC596 -Radiation: Radiotherapy	PTC596 acts as a BMI1 inhibitor by inducing its degradation

## LIST OF ABBREVIATIONS

ACVR1: Activin Receptor Type-1
AFF4: AF4/FMR2 Family Member 4
BET: Bromodomain and Extraterminal
BT: Brain Tumor
BBB: Blood Brain Barrier
CBP: CREB Binding Protein
CCDN1: Cyclin D1
CDK7: Cyclin Dependent Kinase 7
CDK9: Cyclin Dependent Kinase 9
CED: Convection-Enhanced Delivery
CIMP: CpG Island Methylator Phenotype
CNS5: Central Nervous System 5
CSC: Cancer Stem Cell
CTNNB1: Catenin Beta 1
DAC: Decitabine
DIPG: Diffuse Intrinsic Pontine Gliomas
DMG: Diffuse Midline Glioma
DNMTi: DNA Methyltransferase Inhibitors
DZNep: 3-Deazaneplanocin A
EGFR: Epidermal Growth Factor Receptor
EPN: Ependymoma
EZH2: Enhancer of Zeste Homolog-2
ER: Endoplasmic Reticulum
ERBB2: Erb-B2 Receptor Tyrosine Kinase 2
FACT: Facilitates Chromatin Transcription
FGFR: Fibroblast Growth Factor Receptor
GB: Glioblastoma
G34: Glycine 34
HDACi: Histone Deacetylase Inhibitors
HGG: High-Grade Glioma
H3: Histone 3
K27: Lysine 27
H3K27M: H3K27 Mutation
H3F3A: H3 Histone Family 3A
H3K27me3: H3K27 Trimethylation
HIST1H3B: Histone Cluster 1 H3 Family Member B
H3K27ac: H3K27 Acetylation
IDH: Isocitrate Dehydrogenase
IHC: Immunohistochemical
JMJD3: Jumonji Domain-Containing 3
KDM1A: Lysine Demethylase 1A
KDM6A: Lysine Demethylase 6
LSD1: Lysine Specific Histone Demethylase 1
MAO: Monoamine Oxidases
MAPK: Mitogen-Activated Protein Kinase
MB: Medulloblastoma
MYB-L1: MYB-Like 1
NES: Nestin
NSC: Neural Stem Cell
ncRNA: Non-Coding RNA
PDGFΑ: Platelet Derived Growth Factor-Alpha
PF: Posterior Fossa
pHGGs: Pediatric HGGs
PI3K: Phosphatidylinositol 3-Kinase
PTM: Post-Translational Modification
PRC2: Polycomb Repressive Complexes-2
SAHA: Suberoylanilide Hydroxamic Acid
SASP: Senescence Associated Secretory Phenotype
SEC: Super Elongation Complex
ST: Supratentorial
SHH: Sonic Hedgehog
SSRP1: Structure-Specific Recognition Protein 1
SPT16: Suppressor of Ty16
SNAI2: Snail Family Transcriptional Repressor 2
SRY2: SRY-Box Transcription Factor 2
TP53: Tumor Protein 53
UTX: Ubiquitously Transcribed Tetratricopeptide Repeat X Chromosome
WHO: World Health Organization
YAP-1: Yes Associated Protein 1
ZEB1: Zinc Finger E-Box Binding Homeobox 1
ZEB2: Zinc Finger E-Box Binding Homeobox 2
ZFTA: Zinc Finger Translocation Associated
